# Linking childhood emotional abuse and depressive symptoms: The role of emotion dysregulation and interpersonal problems

**DOI:** 10.1371/journal.pone.0211882

**Published:** 2019-02-14

**Authors:** Carolien Christ, Marleen M. de Waal, Jack J. M. Dekker, Iris van Kuijk, Digna J. F. van Schaik, Martijn J. Kikkert, Anna E. Goudriaan, Aartjan T. F. Beekman, Terri L. Messman-Moore

**Affiliations:** 1 Department of Research, Arkin Mental Health Care, Amsterdam, The Netherlands; 2 Department of Psychiatry, GGZ inGeest and Amsterdam UMC, Amsterdam, The Netherlands; 3 Amsterdam UMC, University of Amsterdam, Department of Psychiatry, Amsterdam Institute for Addiction Research, Amsterdam, The Netherlands; 4 Vrije Universiteit Amsterdam, Department of Clinical Psychology, Amsterdam, The Netherlands; 5 Department of Psychology, Miami University, Oxford, Ohio, United States of America; Stellenbosch University, SOUTH AFRICA

## Abstract

Childhood abuse is a major public health problem that has been linked to depression in adulthood. Although different types of childhood abuse often co-occur, few studies have examined their unique impact on negative mental health outcomes. Most studies have focused solely on the consequences of childhood physical or sexual abuse; however, it has been suggested that childhood emotional abuse is more strongly related to depression. It remains unclear which underlying psychological processes mediate the effect of childhood emotional abuse on depressive symptoms. In a cross-sectional study in 276 female college students, multiple linear regression analyses were used to determine whether childhood emotional abuse, physical abuse, and sexual abuse were independently associated with depressive symptoms, emotion dysregulation, and interpersonal problems. Subsequently, OLS regression analyses were used to determine whether emotion dysregulation and interpersonal problems mediate the relationship between childhood emotional abuse and depressive symptoms. Of all types of abuse, only emotional abuse was independently associated with depressive symptoms, emotion dysregulation, and interpersonal problems. The effect of childhood emotional abuse on depressive symptoms was mediated by emotion dysregulation and the following domains of interpersonal problems: cold/distant and domineering/controlling. The results of the current study indicate that detection and prevention of childhood emotional abuse deserves attention from Child Protective Services. Finally, interventions that target emotion regulation skills and interpersonal skills may be beneficial in prevention of depression.

## Introduction

Childhood abuse is widely acknowledged as a major public health problem with detrimental effects on adult mental health (i.e., [1, 2]). Numerous studies have associated childhood abuse with a variety of adverse effects, such as mood and anxiety disorders [1–4] and suicidal behaviors [5–7]. In particular, childhood abuse has consistently been linked to depressive disorders in adulthood in both retrospective studies [3, 8] and prospective studies (e.g., [9, 10]).

The vast majority of studies on childhood abuse have focused on the impact of either childhood sexual abuse (CSA) or childhood physical abuse (CPA) [11], linking both types of abuse to adult depression [12–15]. However, studies that examined the impact of multiple types of abuse have demonstrated childhood emotional abuse (CEA) to be even more strongly related to depression than CSA and CPA [16–19]. CEA refers to an aggressive attitude towards a child, which is not physical in nature, and may include verbal assaults on one’s sense of worth or wellbeing, or any humiliating or demeaning behavior [20]. CEA is far more prevalent than CSA and CPA: recent meta-analyses demonstrated global self-reported prevalence of 127 per 1,000 for CSA [21], 226 per 1,000 for CPA [22], and as high as 363 per 1,000 children for CEA [23].

An important limitation of previous studies on different types of childhood abuse lies in the fact that most did not address the unique impact of each type of childhood abuse, while controlling for the other types. There is substantial overlap and intercorrelation among different types of childhood abuse in community-based samples [2, 24, 25], and this co-occurrence of different types of maltreatment results in a greater likelihood of negative mental health outcomes [24, 25]. Therefore, the unique impact of each type of childhood abuse may be overestimated or distorted by the impact of other types. Hence, it is necessary to examine both the unique and collective impact of all three types of childhood abuse on negative outcomes, while adjusting for the other types. With regard to CEA, only one study examined its impact with adjustment for CSA and CPA, and found that CEA was significantly associated with depressive symptomatology in women [26].

Given the high prevalence of childhood abuse in general, and CEA in particular, it is of utmost importance to examine how they lead to an increased risk of adult depression. Therefore, the main objective of this study is to identify underlying psychological processes that may act as a mediator in this relationship, and may be useful targets for prevention of depression. Several psychological processes have been associated with both general childhood abuse (including CSA, CPA, and CEA) and depression, among which are emotion dysregulation [27] and interpersonal problems [28].

### Emotion dysregulation

Recent evidence suggests that emotion dysregulation plays an important role in the relation between childhood abuse and depression [27]. Emotion regulation refers to “the processes responsible for monitoring, evaluating, and modifying emotional reactions, especially their intensive and temporal features, to accomplish one’s goals” ([29], pp. 27–28). In individuals with current or lifetime major depressive disorder, emotion dysregulation was found to cross-sectionally mediate the relationship between severity of general childhood abuse and depression severity [27, 30]. With regard to CEA, emotion dysregulation cross-sectionally mediated the association between CEA and depressive symptoms in studies among female college students [31], depressed inpatients [32], and low-income African-Americans [33]. The study among college students did not include CSA and CPA, whereas the latter two studies did. For CPA, no evidence was found for a relationship with depression mediated by emotion dysregulation [32, 33]. Regarding CSA, emotion dysregulation mediated the relationship with depression only in the African-American sample [33]. The adverse effect of childhood abuse on emotion dysregulation was found to be specific for CEA: CEA significantly predicted emotion dysregulation above and beyond the effects of CSA and CPA in female college students [34]. Similarly, in another college sample, CEA exclusively predicted difficulties with responding to and dealing with emotions, but not with recognizing and understanding emotions [35]. Hence, the mediating role of emotion dysregulation in the relationship with depression may be largely specific to CEA.

Although it should be noted that cross-sectional mediation provides an indication for, but not proof of the presence of an underlying mechanism, these previous findings suggest that emotion dysregulation contributes to the link between CEA and adult depression [31–33]. However, assessment of emotion dysregulation was limited in these earlier studies. Schulz et al. (2017) used a specific measure of emotion acceptance (i.e., Emotion Acceptance Questionnaire; [36]), rather than overall emotion dysregulation. Crow et al. (2014) used a relatively novel measure of emotion dysregulation with only modest evidence on psychometric properties (i.e., the 12-item version of the Emotion Dysregulation Scale; [37]). Lastly, although Coates and Messman-Moore (2008) used the widely-used Difficulties in Emotion Regulation Scale (DERS; [38]) to measure emotion regulation, they used a latent factor comprised of only two subscales (i.e., Emotional Clarity and Access to Strategies)–thereby omitting other domains of emotion dysregulation captured by the DERS (e.g., lack of emotional awareness and non-acceptance of emotional responses). Therefore, the current study aims to confirm the mediating role of emotion dysregulation in the relationship between CEA and adult depressive symptoms, utilizing a widely-used measure of overall emotion dysregulation (i.e., DERS) with good psychometric properties.

### Interpersonal problems

Although research is still scarce, interpersonal problems have been suggested to be an underlying factor of the link between childhood abuse and depression [39]. Childhood abuse appears to negatively influence social relationships and interpersonal functioning, which was found for CPA [6], CSA [40, 41], and CEA [41]. However, one study in college students found no direct relationship with problems with social relationships for CEA and CPA, whereas CSA was associated with decreased social problems [35]. Individuals with a history of childhood abuse in general reported lower levels of social support in adulthood [42], more dysfunctional relationships, and an increased risk for divorce compared to others [43]. People with a history of childhood abuse have not only reported more difficulties in marital and social relationships than others, but also more interpersonal problems as measured with the widely-used Inventory of Interpersonal Problems (IIP) [44]. The IIP measures persistent difficulties that individuals experience in social relationships, and distinguishes different domains of interpersonal problems (e.g., being domineering/controlling, nonassertive, or self-sacrificing) [44, 45]. Victims of CSA reported more interpersonal difficulties than non-victims on all domains of the IIP [40]. Similarly, in a Korean sample of patients with depression and anxiety disorders, both CEA and CSA, but not CPA, were associated with interpersonal problems as measured with the IIP total score [41]. However, an important limitation of these studies lies in the fact that they did not address the unique impact of each type of childhood abuse, controlled for the other types. Therefore, the unique and collective impact of all three types of childhood abuse on interpersonal functioning remains unknown.

Interpersonal problems have not only been linked to childhood abuse, but also to depression. Recurring frustrated interpersonal dynamics may make an individual more vulnerable to depression [45]. For example, social isolation, avoidance, and submissiveness have been associated with depression [46, 47]. However, it remains unknown whether interpersonal problems mediate the link between CEA and depression, and which specific interpersonal styles as measured with the IIP play a role in this relationship. In the aforementioned study of Whiffen, Thompson and Aube [40], difficulties with being too controlling, being intimate, and being submissive cross-sectionally and partly mediated the relation between CSA and depression in women. Hence, their results suggest that these interpersonal styles partly account for the risk of depression in women with a history of CSA. Interestingly, different interpersonal styles (i.e., being unassertive and taking too much responsibility) partially mediated this relation in men [40].

Previous research has reported a gender difference regarding the associations among childhood abuse, interpersonal relationships, and depression as well. A history of childhood abuse negatively influenced women’s perceived quality of their romantic relationships, whereas this did not hold for men [43]. Furthermore, emotionally supportive social relationships were shown to be substantially more protective against a depressive disorder for women than for men [48]. Lastly, the effect of CEA on internalizing symptoms (i.e., peer problems and emotional symptoms) and mental well-being was larger for girls than for boys in a Swedish population sample [49]. Hence, although the majority of research found no gender differences regarding the prevalence of CEA [1, 23], previous findings suggest that its effect on interpersonal functioning, emotion regulation, and depression may be stronger for women compared to men. Therefore, also given women’s substantially increased risk to develop a major depressive disorder compared to men [50], this study is conducted in a female sample.

### Aims

In the current study, our aims were threefold. First, we examined the collective and unique impact of childhood emotional abuse, physical abuse, and sexual abuse on depressive symptoms, emotion dysregulation, and interpersonal problems in a European sample of female college students. Based on current literature [26, 34], we hypothesized that only emotional abuse would be independently associated with depressive symptoms and emotion dysregulation, if the three types of childhood abuse were controlled for each other. Furthermore, we hypothesized that all three types of childhood abuse would be independently associated with interpersonal problems. Second, if evidence for a relationship between CEA and depression indeed existed, as hypothesized, we aimed to examine whether this relationship would be cross-sectionally mediated by emotion dysregulation and interpersonal problems. We hypothesized that both emotion dysregulation and interpersonal problems would mediate the relationship between CEA and depressive symptoms. Finally, we aimed to explore whether specific domains of interpersonal problems could be identified as particularly important in explaining the relationship between CEA and depressive symptoms.

## Materials and methods

### Design and procedures

This cross-sectional study was conducted at the VU University in Amsterdam, The Netherlands, in collaboration with the research department of Arkin. The data collection took place in April and May 2017. College students were recruited by research assistants through flyers posted on campus, Facebook, and online research recruitment platforms. All participants provided written informed consent prior to participation. Participants completed a set of self-report questionnaires with forced responses administered in Net-Q: an online secured survey platform. Each assessment was supervised by one of two research assistants (MSc or BSc) and took approximately 45 minutes. Participants were compensated with €10 or 50 minutes course credit.

### Participants

The inclusion criteria were: being female, being currently studying in the Netherlands, and having sufficient understanding of the Dutch language to administer self-report questionnaires. The study sample consisted of 276 female college students with a mean age of 21.7 years (*SD* = 2.38). As shown in Table 1, participants were predominantly born in the Netherlands (91.7%), single (66.3%), living with their parents (42.0%) or with roommates (35.1%), and studying psychology (50.4%).

**Table 1:** Demographic characteristics of the total study sample (*N* = 276).

	*N*	%
Country of birth		
Netherlands	253	91.7
Other European country	12	4.3
Non-European country	11	4.0
Country of birth of parents		
Both Netherlands	192	69.6
Both Europe	21	7.6
One Netherlands, one non-European	19	6.9
Both non-European	44	15.9
Relationship status		
Single	183	66.3
In a relationship	93	33.7
Living situation		
With parent(s)/caregiver(s)	116	42.0
With roommates	97	35.1
Alone	36	13.0
With partner	26	9.4
Other	1	0.3
Field of study		
Psychology	139	50.4
Pedagogical and other social sciences	41	14.9
Medicine/health sciences	24	8.7
Management/business/economics	23	8.3
Communication science	15	5.4
Law	12	4.3
Natural/formal sciences	11	4.0
Other	11	4.0

### Measures

#### Demographics

The demographic characteristics age, country of birth, country of birth of parents, relationship status, living situation, and field of study were collected during the self-report assessment.

#### Childhood abuse

The short form of the Childhood Trauma Questionnaire (CTQ-SF) [51, 52] was used to assess severity of CEA (i.e., verbal assaults on one’s sense of worth or wellbeing, or any humiliating or demeaning behavior), CPA (i.e., bodily assaults that posed a risk of or resulted in injury), and CSA (i.e., unwanted sexual contact or conduct). Each scale consists of 5 items, rated on a 5-point, Likert-type scale with response options ranging from “Never true” to “Very often true”, with the exception of the CSA scale. Previous psychometric analyses of the Dutch version indicated that one item of the CSA scale (“During my youth, I was molested by someone”) had to be removed [52]. A total score was calculated for each scale by summing all items. The sum score of the CSA scale was multiplied by 5/4 to enable comparisons with other studies using the CTQ-SF. The sum scores of the scales were used as continuous variables to address the main research questions. The CTQ-SF manual provides cut-off scores to describe presence/absence of abuse and levels of abuse severity (none, mild, moderate, severe) for each type of childhood abuse [51]; in the current study, these cut-offs were used for descriptive purposes only. Validity and reliability of the Dutch version of the CTQ-SF are good [52, 53]. In the current study, internal consistency was good for the CEA scale (α = .80), acceptable for the CPA scale (α = .76), and excellent for the CSA scale (α = .91).

#### Depressive symptoms

Depressive symptoms were measured with the Quick Inventory of Depressive Symptoms (QIDS-SR-16), a 16-item self-rated depression severity scale [54]. The questionnaire rates the severity of the DSM-IV criteria for depressive disorder [55] in the preceding seven days. Total scores range from 0 to 27. A systematic review of the psychometric properties of the QIDS-SR-16 reported that the internal consistency was acceptable (α = .69 to .89), and concurrent validity with other depression scales was moderate to high [56]. In the current study, the internal consistency of the QIDS-SR-16 was acceptable (α = .76).

#### Emotion dysregulation

Emotion dysregulation was measured with the Difficulties in Emotion Regulation Scale (DERS) [38, 57]. The DERS is a 36-item self-report questionnaire that evaluates emotion regulation difficulties across multiple domains: non-acceptance of emotional responses, difficulties engaging in goal-directed behavior, impulse control difficulties, lack of emotional awareness, limited access to emotion regulation strategies, and lack of emotional clarity. Total scores range from 36 to 180. The DERS showed high internal consistency and good test-retest reliability in a student sample [38]. In the current study, the internal consistency of the DERS was excellent (α = .93).

#### Interpersonal problems

Interpersonal difficulties were measured with the Inventory of Interpersonal Problems (IIP-32): a self-report questionnaire with good internal consistency and test-retest reliability [44]. The IIP-32 consists of 32 items and comprises eight subscales related to different domains of interpersonal problems, which showed acceptable to good internal consistency in the current study: domineering/controlling (α = .68), vindictive/self-centered (α = .78), cold/distant (α = .76), socially inhibited (α = .80), nonassertive (α = .77), overly accommodating (α = .71), self-sacrificing (α = .72), and intrusive/needy (α = .74).

### Ethics

The study was reviewed and approved by the ethics committee of the VU University, Amsterdam, The Netherlands (VCWE-2017-009R1). Participation in the study was voluntary, and all participants provided written informed consent.

### Statistical analyses

Data were analyzed using SPSS Statistics 24.0. There were no missing values. First, univariate linear regression analyses were performed to examine associations between the continuous variables CEA, CPA, and CSA, and the outcome variables depressive symptoms (QIDS-SR-16), emotion dysregulation (DERS total score), and interpersonal problems (IIP-32 total score). Subsequently, three multiple linear regression analyses were performed in order to determine which types of childhood abuse were independently associated with these outcome variables. Assumptions for regression analyses (linearity, homoscedasticity, normality of the residuals, absence of multicollinearity) were met.

If there was evidence for an association between CEA and depressive symptoms, we proceeded with conducting mediation analyses. To test the potential mediation effects of emotion dysregulation (DERS total score) and interpersonal problems (IIP-32 total score), we performed two simple mediation analyses using the PROCESS macro [58] for SPSS, which is based on OLS regression analysis. Rijnhart, Twisk, Chinapaw, de Boer and Heymans [59] demonstrated that when analyzing simple mediation models with a continuous mediator and continuous outcome variable, OLS regression yields the same results as SEM and the potential outcomes framework. Finally, to explore whether specific domains of interpersonal problems could be identified as particularly important in explaining the relationship between CEA and depressive symptoms, we performed a multiple mediation analysis including all eight IIP-32 subscales as mediator variables. The presence and magnitude of mediation was determined by estimating the indirect effects. To test the statistical significance of the indirect effects, we used bias-corrected 95% bootstrap confidence intervals (based on 5000 bootstrap samples) as calculated by PROCESS [58]. If zero was not contained in the confidence interval, we concluded that the indirect effect was significant. To measure the mediation effect size, we used P_M_: the proportion of each indirect effect relative to the total effect. An alpha level of 0.05 and confidence interval of 95% were used in all analyses.

## Results

### Descriptive statistics

The severity levels for each type of childhood abuse are shown in Table 2. Using the lowest CTQ-SF cut-off scores, we found that 31.5% of the participants reported CEA, 8.0% reported CPA, and 11.6% reported CSA. Descriptive statistics of the self-report measures of severity of childhood abuse, depressive symptoms, emotion dysregulation, and interpersonal problems are presented in Table 3. The CTQ-SF subscales showed considerable correlations. The highest correlation was found between CEA and CPA (Pearson r = .49), whereas lower correlations were found between CEA and CSA (Pearson r = .37), and CPA and CSA (Pearson r = .27). Using the QIDS-SR scoring guidelines, 59.8% of the participants reported no depressive symptoms (score 0–5), 30.1% reported mild depressive symptoms (score 6–10), 8.0% reported moderate depressive symptoms (score 11–15), and 2.1% reported (very) severe depressive symptoms (score 16+) [54]. The highest IIP-32 subscale scores were found for the domains overly accommodating, self-sacrificing, and nonassertive, whereas the lowest subscale scores were found for vindictive/self-centered, cold/distant, and domineering/controlling. This is in accordance with the female norm group described in the IIP manual [44].

**Table 2:** Severity levels of three types of childhood abuse in female college students (*N* = 276).

Severity level (CTQ-score)	*N*	%
CEA		
None (5–8)	189	68.5
Mild (9–12)	57	20.7
Moderate (13–15)	19	6.9
Severe (16+)	11	4.0
CPA		
None (5–7)	254	92.0
Mild (8–9)	9	3.3
Moderate (10–12)	9	3.3
Severe (13+)	4	1.4
CSA		
None (5)	244	88.4
Mild (6–7)	13	4.7
Moderate (8–12)	10	3.6
Severe (13+)	9	3.3

*Note*. CEA = childhood emotional abuse; CPA = childhood physical abuse; CSA = childhood sexual abuse.

**Table 3:** Descriptive statistics of the self-report measures of severity of childhood abuse, depressive symptoms, emotion dysregulation, and interpersonal problems in female college students (*N* = 276).

	*M*	*SD*	Range
Childhood emotional abuse (CTQ-CEA)	7.8	3.4	5–22
Childhood physical abuse (CTQ-CPA)	5.7	1.8	5–18
Childhood sexual abuse (CTQ-CSA)	5.7	2.6	5–21
Depressive symptoms (QIDS-SR)	5.4	4.0	0–22
Emotion dysregulation (DERS)	84.9	19.9	44–139
Interpersonal problems (IIP-32 total)	34.6	13.9	3–78
Vindictive/self-centered (IIP-32 subscale)	2.4	2.5	0–15
Cold/distant (IIP-32 subscale)	2.6	2.6	0–11
Socially inhibited (IIP-32 subscale)	3.6	3.1	0–14
Nonassertive (IIP-32 subscale)	5.5	3.3	0–16
Overly accommodating (IIP-32 subscale)	6.6	3.4	0–16
Self-sacrificing (IIP-32 subscale)	6.6	3.2	0–15
Intrusive/needy (IIP-32 subscale)	4.3	3.1	0–15
Domineering/controlling (IIP-32 subscale)	3.0	2.6	0–13

*Note*. CEA = childhood emotional abuse; CPA = childhood physical abuse; CSA = childhood sexual abuse; QIDS-SR = Quick Inventory of Depressive Symptomatology; DERS = Difficulties in Emotion Regulation Scale; IIP-32 = Inventory of Interpersonal problems.

### Univariate linear regression analyses

Table 4 provides the results of the univariate linear regression analyses for examining the association between three types of childhood abuse and depressive symptoms, emotion dysregulation, and interpersonal problems. CEA was significantly associated with depressive symptoms (*b* = 0.38, *t* = 5.72, *p* < .001, *R*^*2*^ = .11), emotion dysregulation (*b* = 1.14, *t* = 3.33, *p* = .001, *R*^*2*^ = .04), and interpersonal problems (*b* = 1.13, *t* = 4.81, *p* < .001, *R*^*2*^ = .08). CPA was significantly associated with depressive symptoms (*b* = 0.33, *t* = 2.45, *p* = .015, *R*^*2*^ = .02) and interpersonal problems (*b* = 1.23, *t* = 2.66, *p* = .008, *R*^*2*^ = .03). No other significant associations between types of childhood abuse and outcome variables existed.

**Table 4:** Results of univariate linear regression analyses for predicting depressive symptoms, emotion dysregulation, and interpersonal problems in female college students (*N* = 276).

	QIDS-SR		DERS		IIP-32	
	*B (SE)*	*p*	*B (SE)*	*p*	*B (SE)*	*p*
CEA	0.38 (0.07)	**< .001**	1.14 (0.34)	.**001**	1.13 (0.24)	**< .001**
CPA	0.33 (0.13)	.**015**	0.80 (0.67)	.235	1.23 (0.46)	.**008**
CSA	0.09 (0.09)	.338	0.21 (0.46)	.640	0.49 (0.32)	.128

*Note*. CEA = childhood emotional abuse; CPA = childhood physical abuse; CSA = childhood sexual abuse; QIDS-SR = Quick Inventory of Depressive Symptomatology; DERS = Difficulties in Emotion Regulation Scale; IIP-32 = Inventory of Interpersonal problems.

P-values indicating significance at the .05 level are shown in bold.

### Multivariate linear regression analyses

The multiple linear regression analyses revealed significant overall models for depressive symptoms (*F*(3,272) = 11.34, *p* < .001, *R*^*2*^ = .11), emotion dysregulation (*F*(3,272) = 3.96, *p* = .009, *R*^*2*^ = .04), and interpersonal problems (*F*(3,272) = 7.73, *p* < .001, *R*^*2*^ = .08). All variance inflation factors (VIFs) were less than 1.5, indicating that multicollinearity was not present. As shown in Table 5, emotional abuse was the only form of childhood abuse that was independently associated with depressive symptoms, with emotion dysregulation, and with interpersonal problems.

**Table 5:** Results of multiple linear regression analyses for predicting depressive symptoms, emotion dysregulation, and interpersonal problems in female college students (*N* = 276).

	QIDS-SR		DERS		IIP-32	
	*B* [99% CI]	*p*	*B* [99% CI]	*p*	*B* [99% CI]	*p*
CEA	0.42 [0.26, 0.57]	**< .001**	1.33 [0.52–2.14]	.**001**	1.09 [0.54–1.65]	**< .001**
CPA	-0.03 [-0.32, 0.27]	.866	-0.32 [-1.82–1.19]	.681	0.23 [-0.80–1.26]	.662
CSA	-0.11 [-0.29, 0.08]	.254	-0.37 [1.32–0.59]	.452	-0.08 [-0.74–0.58]	.809

*Note*. CEA = childhood emotional abuse; CPA = childhood physical abuse; CSA = childhood sexual abuse; QIDS-SR = Quick Inventory of Depressive Symptomatology; DERS = Difficulties in Emotion Regulation Scale; IIP-32 = Inventory of Interpersonal problems.

P-values indicating significance at the .05 level are shown in bold.

### Mediation analyses

**Fig 1:**
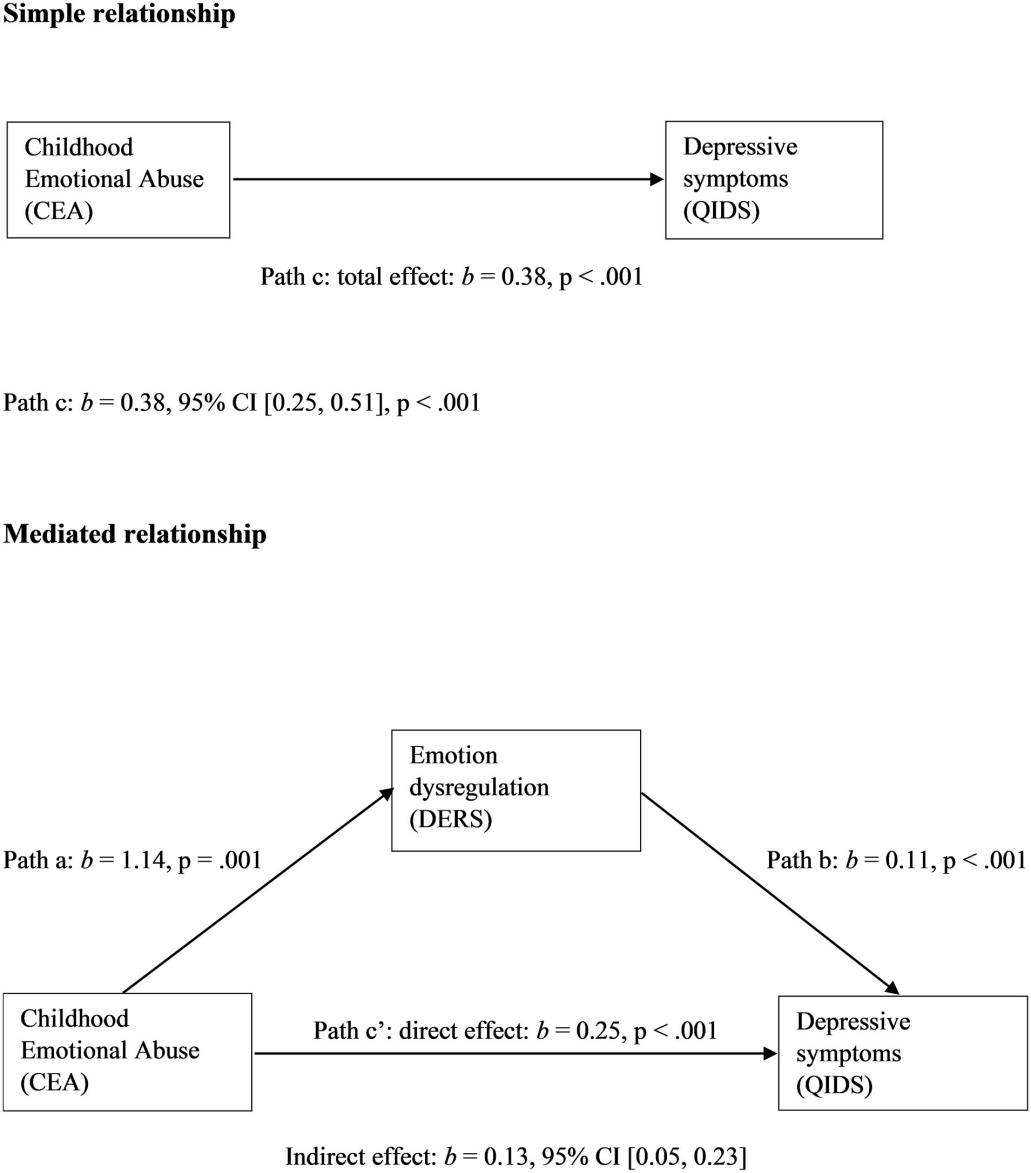
Model of childhood emotional abuse as predictor of depressive symptoms, mediated by emotion dysregulation.

**Fig 2:**
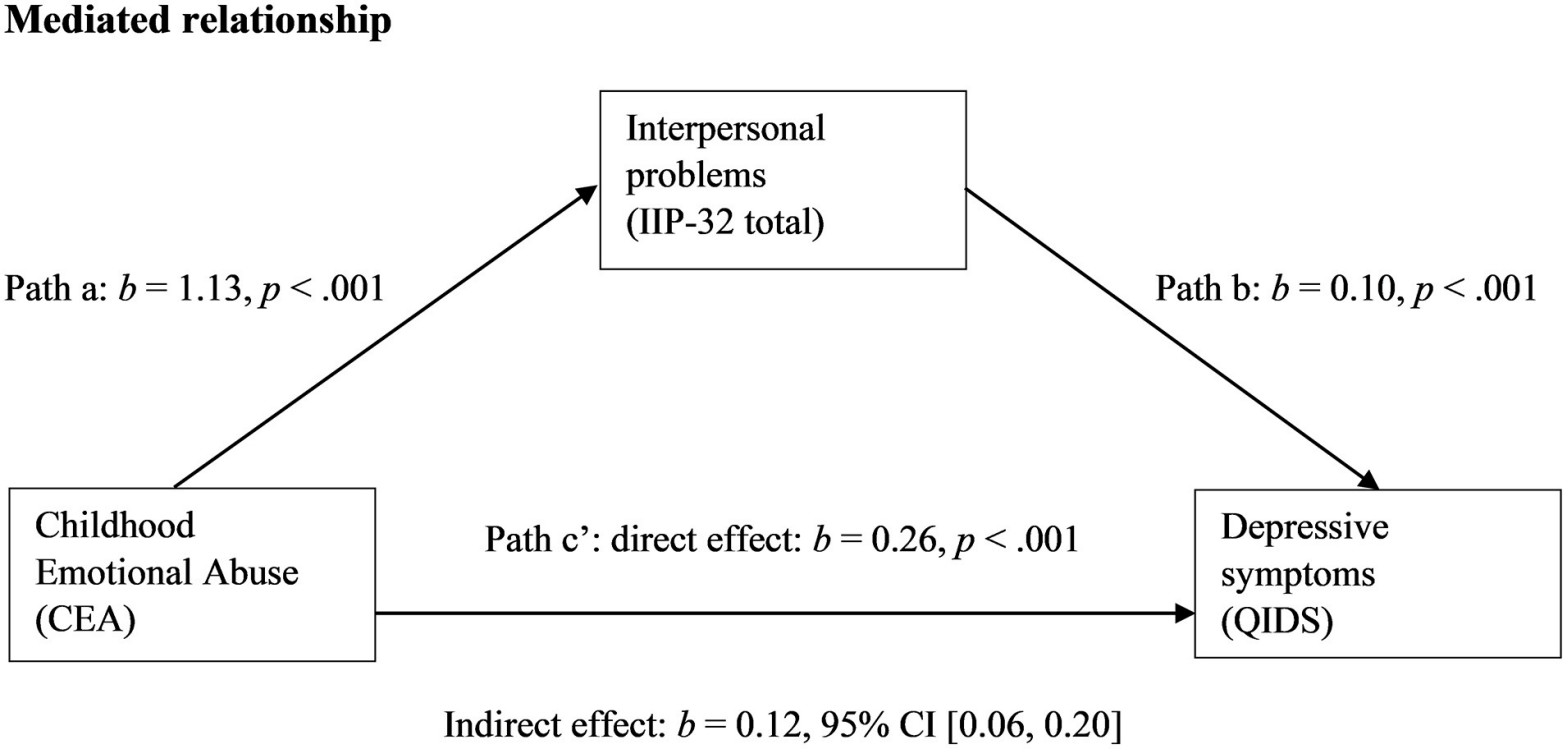
Model of childhood emotional abuse as predictor of depressive symptoms, mediated by interpersonal problems.

Emotion dysregulation significantly mediated the effect of CEA on depressive symptoms in a simple mediation model, as displayed in Fig 1. The indirect effect (*b* = 0.13) explained 34% of the total effect (*b* = 0.38). Additionally, interpersonal problems significantly mediated the effect of CEA on depressive symptoms in a separate simple mediation model, as displayed in Fig 2. The indirect effect (*b* = 0.12) explained 31% of the total effect (*b* = 0.38).

Table 6 provides the results of the multiple mediation analysis for the eight domains of interpersonal problems. The effect of CEA on depressive symptoms was significantly mediated by the following domains of interpersonal problems: cold/distant (proportion mediated: 9%) and domineering/controlling (proportion mediated: 14%). Although the domain self-sacrificing was significantly associated with depressive symptoms, it was not significantly associated with CEA.

**Table 6:** Results of multiple mediation analysis for testing all eight domains of interpersonal problems as mediators of the effect of childhood emotional abuse on depressive symptoms in female college students (*N* = 276).

Mediators (M)	*b* of CEA → M (path a)	*b* of M → QIDS (path b)	*b* of indirect effect (a x b) [95% CI]	*P*_*M*_: proportion of indirect effect of total effect [95% CI]
Vindictive/self-centered	0.12**	-0.05	-0.01 [-0.04, 0.02]	-0.02 [-0.12, 0.05]
Cold/distant	0.15**	0.23*	**0.03**^a^ [0.01, 0.08]	0.09 [0.01, 0.23]
Socially inhibited	0.19***	0.10	0.02 [-0.02–0.08]	0.05 [-0.05, 0.21]
Nonassertive	0.12*	0.12	0.01 [-0.01, 0.07]	0.04 [-0.02, 0.17]
Overly accommodating	0.11	0.07	0.01 [-0.02, 0.05]	0.02 [-0.05, 0.14]
Self-sacrificing	0.11	0.19*	0.02 [-0.00, 0.07]	0.05 [-0.00, 0.18]
Intrusive/needy	0.15**	-0.09	-0.01 [-0.05, 0.01]	-0.04 [-0.15, 0.02]
Domineering/controlling	0.18***	0.28**	**0.05**^a^ [0.01, 0.12]	0.14 [0.03, 0.33]

^a^ = significant at the .05 level: 95% bias-corrected bootstrap confidence interval did not contain zero.

* *p* < .05.

** *p* < .01.

*** *p* < .001.

## Discussion

The first aim of this study was to examine whether CEA, CPA, and CSA are independently associated with depressive symptoms, emotion dysregulation, and interpersonal problems in female college students. As hypothesized, CEA was independently associated with depressive symptoms and emotion dysregulation, whereas CPA and CSA were not. These results are consistent with previous studies that found CEA to be more strongly related to depression than CSA and CPA [10, 16–19, 26]. In addition, these findings are in line with research demonstrating that CEA significantly predicted emotion dysregulation above and beyond the effects of CSA and CPA in female college students [34] and inpatients with substance use disorders [60]. An explanation for this consistently reported result may be found in children’s development of emotion regulation skills, which is highly influenced by the family emotional climate and interactions with primary caregivers [61, 62]. A child’s emotion regulation capabilities can be undermined by parents’ denigrating or dismissive behaviors and frequent negative emotions directed at the child [61, 63]. These patterns appear to be most exemplary for CEA, as opposed to CPA and CSA. Moreover, children are assumed to imitate their parents’ modes of emotion regulation. Poor emotion regulation modeling by primary caregivers in the form of CEA may lead to emotion regulation deficits in children [64].

Contrary to our expectations, only CEA was independently associated with interpersonal problems—although CPA was significantly associated with interpersonal problems at the univariate level. These findings are inconsistent with previous studies that demonstrated CSA to be associated with interpersonal problems [40, 41]. However, no previous study has examined multiple types of childhood abuse in one model, thereby examining the unique impact of each type, while adjusting for the other types. Our results suggest that only CEA contributes to interpersonal problems above and beyond the impact of CSA and CPA. CEA involves humiliating, demeaning behavior and verbal assaults on one’s sense of worth or well-being [51], which, together with the often more chronic character of CEA compared to CPA and CSA, are likely to influence one’s attitudes and behavior towards others.

Additionally, we aimed to examine whether the relationship between CEA and depressive symptoms is mediated by emotion dysregulation and interpersonal problems. As hypothesized, we found that both emotion dysregulation and interpersonal problems mediated this relationship. With regard to emotion dysregulation, this finding is consistent with previous research in samples of female college students [31], low-income African-Americans [33], and depressed inpatients [32]. The current study adds to the literature by confirming the mediating role of emotion dysregulation in the association between CEA and depressive symptoms in a European sample of female college students, and by using a widely-used measure of overall emotion dysregulation with good psychometric properties.

With regard to interpersonal problems, our study is the first to identify interpersonal problems as a mediator of the association between CEA and depressive symptoms. This study extends previous research that associated CEA with interpersonal problems [41]. Possibly, individuals who report a higher level of interpersonal problems experience less social support. It has consistently been shown that social support and larger, diverse social networks are protective against depression in the general population [46, 65], especially in women [48]. Importantly, perceived social support, rather than received social support plays an important protective role against depression in the general population (see [46], for a review). Correspondingly, in patients with depressive and anxiety disorders, perceived social disability, rather than loneliness or a low level of social activities, predicted persistence of these disorders at 2-year follow-up [66].

Our final aim was to explore whether specific domains of interpersonal problems could be identified as particularly important in explaining the relationship between CEA and depressive symptoms. We found that this relationship was mediated by cold/distant and domineering/controlling interpersonal styles. Cold/distant individuals do not feel close to or loving toward others, and find it hard to make and maintain long-term commitments to other people [44]. Children suffering from emotional abuse by attachment figures may develop negative internal working models of the self and others that interfere with social functioning as the child matures, and can lead to interpersonal avoidance and a lack of trust in others [67, 68]–possibly reflecting a cold/distant interpersonal style. Avoidance of social situations and being cold/distant can in turn lead to depression [47, 69].

Domineering/controlling individuals describe themselves as too controlling or manipulative, and attempt to influence others by arguing excessively and sometimes showing hostile or aggressive behavior [44]. Our findings are in line with Huh, Kim, Yu and Chae [41], who reported that CEA is associated with being more domineering/controlling in a sample of adult outpatients with depression and anxiety disorders. To the best of our knowledge, no previous study reported an association between domineering/controlling behavior as measured with the IIP [44] and depressive symptoms. However, Barrett and Barber [47] compared interpersonal profiles of adults with major depressive disorder with the IIP-normative sample, and found no significant differences between these groups on the subscale domineering/controlling. Future prospective studies are needed to examine whether domineering/controlling behavior stems from CEA, and to what extent this type of interpersonal problems may cause a higher risk of depression.

### Strengths and limitations

The current study has several strengths. This study is the first to explore the mediating role of interpersonal problems in the association between CEA and depressive symptoms. In addition, this study is the first to confirm previous research indicating emotion dysregulation to be a mediator in the association between CEA and depressive symptoms in a European sample of college students, using well-validated measures. Furthermore, we determined the unique and collective impact of three types of childhood abuse in multiple regression models, whereas the majority of previous studies only addressed a single type of childhood abuse.

This study also has some limitations. First, since this is a cross-sectional study, we cannot draw firm conclusions on causality − therefore, our results should be interpreted with caution. Longitudinal research is needed to confirm that emotion dysregulation and interpersonal functioning prospectively mediate the effect of CEA on depression and do indeed represent causal mechanisms. Second, the study relied exclusively on self-report measures; however, all measures have good psychometric properties and are widely-used. The DERS is based on a clinical-contextual model of emotion regulation [38], which has been embraced within applied clinical research, but differs from the more narrow conceptualization of emotion regulation in studies in the field of affective science (e.g., [70]), which focus on the process of regulating emotions without including broader trait-like abilities such as clarity and self-control. With regard to the Childhood Trauma Questionnaire, both the full scale and subscales were demonstrated to remain stable after 6 months of treatment despite significantly reduced psychopathology [71]. Third, the prevalence of CPA (*N* = 22, 8.0%) and CSA (*N* = 34, 13.0%) was relatively low, which may limit the statistical power to detect possible weak associations between these types of abuse and the outcome variables. Fourth, the generalizability of our results may be limited, since our sample consisted of well-educated, young adult women. However, the association between childhood maltreatment and adult depression did not differ between men and women in a large sample of patients [72]. Lastly, as the majority of our non-clinical sample reported none to mild depressive symptoms, results are not generalizable to depressed patients. However, well-educated, young adult women are an important high-risk group for developing a depression [50, 73]. A recent survey of the WHO World Mental Health International College Student project among 8 countries demonstrated depressive disorder to be the most common mental disorder in first-year college students, with a 12-month prevalence of 18.5% [74]. Importantly, female college students were at increased risk for both lifetime and 12-month mental disorders compared to male students [74].

### Recommendations

Our findings indicate that both emotion dysregulation and specific types of interpersonal problems play an important role in the relationship between CEA and depression in female college students. Future studies should examine the collective and unique impact of different types of childhood abuse on specific types of interpersonal problems in diverse samples, such as lower educated samples, different age groups, males, and clinical samples of patients with mental disorders. Furthermore, our findings should be replicated in prospective studies that are able to examine whether emotion dysregulation and interpersonal problems temporally precede the emergence of depressive symptoms. In addition, future studies should further examine the relation between interpersonal problems and depression, and the potential role of social support in this relationship.

The current study indicates that detecting and preventing CEA deserves as much attention from Child Protective Services as CPA and CSA. If emotion dysregulation and specific types of interpersonal problems are replicated in future studies as crucial links between CEA and depressive symptoms, prevention programs that target these specific, underdeveloped skills in children and adults with a history of CEA may be beneficial in primary prevention of depression. Furthermore, clinicians should recognize and acknowledge the harmful impact of CEA in treatment of depressed individuals. Incorporating treatment techniques aimed at strengthening emotion regulation and interpersonal skills (e.g., [75, 76]) may enhance treatment outcome and reduce the risk of relapse in depressed patients with a history of CEA.
